# ^125^I Radioactive Seeds Implantation Therapy for Hepatocellular Carcinoma

**DOI:** 10.4021/gr2009.05.1289

**Published:** 2009-05-20

**Authors:** Jin Lv, Xiu Feng Cao, Bin Zhu

**Affiliations:** a Oncology Center, Department of Surgery, Nanjing First Hospital Affiliated to Nanjing Medical University, Nanjing 210006, Jiangsu Province, China

**Keywords:** ^125^I, Brachytherapy, Hepatocellular carcinoma, Transcatheter arterial chemoembolization

## Abstract

**Background:**

This study was to evaluate the outcome and the prognostic factors of unresectable hepatocellular carcinoma (HCC) patients with ^125^I radioactive seeds implantation, who had failed transcatheter arterial chemoembolization (TACE).

**Methods:**

From September 2002 to March 2006, 48 patients with unresectable HCC underwent ^125^I permanent implantation brachytherapy. Thirty-eight patients were male and 10 were female. Mean age was 59 years, ranginging from 32 to 86. Karnofsky performance status(KPS) was 100 in 10 patients, 80 in 21 patients, and 60 in 17 patients. According to Child-Pugh classification of liver, 34 patients were in class A and 14 patients in class B. Twenty-two patients had alpha-fetoprotein (AFP) level > 400 ng/ml. Tumor size was < 5cm in 17 patients, 5-10 cm in 18 patients, and > 10cm in 13 patients. Thirty-four patients had confluent tumors, 14 patients presented single hepatic tumor. Serum hepatitis antigen markers were positive for type B in 38 patients and type C in 10 patients. Twenty-two patients had Okuda Stage I, 24 patients Stage II, and 2 patients Stage III. According to the AJCC staging system (6th edition), 10 patients were in Stage II (T2N0M0), 20 in Stage IIIa (T3N0M0) and 18 in Stage IIIb (T4N0M0).

**Results:**

An objective response was observed in 34 of 48 patients, giving a response rate of 70.8%. The survival rates at 1, 2 and 3 years were75%, 45.8% and 27.1%, respectively. In the analysis of prognostic factors, tumor type, tumor size, Okuda stage, AJCC stage, Liver Child-Pugh, pretreatment AFP level, and matched peripheral dose (MPD) all had significant impact on survival.

**Conclusions:**

The^ 125^I permanent implantation brachytherapy induced a substantial tumor response rate of 70.8% with survival rates at 1, 2 and 3 years of 75%, 45.8% and 27.1%, respectively, and a median survival time of 15.5 months in patients with unresectable HCC who had failed TACE. The complications are acceptable and can be managed with conservative treatment. Although we do not know whether there is a survival benefit through the use of this treatment,^ 125^I permanent implantation brachytherapy seems to be a practical method of salvage for this subset of patients. Further study is warranted to evaluate the survival of such patients with controlled trial.

## Introduction

Hepatocellular carcinoma (HCC) is one of the most common malignancies and the third most common cause of cancer death worldwide, with an overall five-year survival rate of approximately 5% [1]. Surgical resection and transplantation have been considered the optimal therapies for long-term control of the disease. However, most patients with HCC present with advanced disease, only 10% to 20% of patients suitable for surgical intervention due to multiple tumors, inadequate liver function, and/or involvement of vascular or major biliary structures. In many trials, transcatheter arterial chemoembolization (TACE) had been shown to improve survival compared to best supportive care in patients with unresectable HCC [2, 3]. However, its benefit is modest and limited. Therefore, it should be considered for combination with other adjuvant treatment. In recent 20 years, HCC belonging to the radiosensitive tumor had been confirmed. With the development of the new radiotherapy technology and facility, the research about brachyhtherapy, especially ^125^I seeds implantation therapy, has provoked more interests in the world. The purpose of this study was to evaluate the outcome and the prognostic factors of unresectable HCC patients, who failed the TACE, treated with ^125^I radioactive seeds implantation therapy.

## Materials and Methods

### Patients

All patients signed the informed consent prior to their inclusion in the study, which had been reviewed by the appropriate ethics committee and had been performed in accordance with the ethical standards laid down in an appropriate version of the 1964 Declaration of Helsinki. From September 2002 to March 2006, 58 patients with unresectable HCC were referred, in Oncology Center Surgery of Nanjing First Hospital, for ^125^I seeds implantation. These patients had failed TACE. No patient had received prior radiotherapy for liver disease. Ten patients were excluded owing to Child-Pugh class C or Karnofsky performance status (KPS) < 50. The diagnosis of HCC was based on histological confirmation or on radiography (by CT scan as well as hepatic angiography) and a serum alpha-fetoprotein (AFP) value > 400 ng/ml. Those patients with AFP level < 400 ng/ml underwent liver biopsy for diagnosis. The judgment of TACE failure was based on tumor progression demonstrated on computerized tomography (CT) scan after several sessions. The frequency of TACE was 2-6 sessions (median 4) and time interval between the last TACE and the start of intrahepatic ^125^I brachytherapy was 4 weeks. Patients’ characteristics are shown in Table 1. Thirty-eight patients were male and 10 female. Mean age was 59 years, ranging from 32 to 86. KPS was 100 in 10 patients, 80 in 21 patients, and 60 in 17 patients. According to Child-Pugh classification for cirrhosis of the liver, 34 patients were in class A and 14 patients in class B. Twenty-two patients had AFP level > 400 ng/ml. Tumor size was defined as the mean of three diameters on CT scan, < 5cm in 17 patients, 5-10 cm in 18 patients, and > 10cm in 13 patients. Thirty-four patients had massive tumors, 14 patients presented single hepatic tumor. The percentage of positive HBV/HCV patient was 79.2% and 20.8%, respectively. Twenty-two patients had Okuda Stage I, 24 patients Stage II, and 2 patients Stage III. According to the AJCC staging system (6th edition), 10 patients were in Stage II (T2N0M0), 20 in Stage IIIa (T3N0M0) and 18 in Stage IIIb (T4N0M0).

**Table 1:** Patient characteristics (n = 48)

Characteristic	Number of patients(%)
Age (mean)	32-86 years (59)
Gender	
Male (%)	38(79.2%)
Female (%)	10(20.8%)
KPS	
100	10(20.8%)
80	10(20.8%)
60	17(35.4%)
Viral antigen	
HBV	38(79.2%)
HCV	10(20.8%)
AFP (ng/ml)	
> 400	22(45.8%)
≤ 400	26(54.2%)
Tumor Size	
< 5 cm	17(35.4%)
5-10 cm	23(47.9%)
> 10 cm	8(16.7%)
Tumor type	
Single	14(29.2%)
Massive	34(70.8%)
Liver Child-Pugh	
A	34(70.8%)
B	14(29.2%)
Okuda stage	
I	22(45.8%)
II	24(50%)
III	2(4.2%)
AJCC stage	
II	10(20.8%)
IIIa	20(41.7%)
IIIb	18(37.5%)
Prior TACE (%)	
2	5(10.4%)
2+	43(89.6%)
TACE median sessions	4(95%CI,4-5)

### Implantation brachytherapy planning

A treatment-planning CT scan was performed that included a portion of the inferior chest and the entire abdomen to allow for planning of non-axial fields. The gross tumor volume (GTV) was defined as high CT value area in early phase contrast-enhanced CT images. The clinical target volume (CTV) was defined as the GTV plus 1 cm. The planning target volume (PTV) was defined as the CTV plus 0.5 cm for daily patient setup variation, and 1cm in the cranial-caudal dimension to account for the ventilatory motion of the liver. Treatment plans were designed for each patient with the high-dose region including the PTV. The postplans were evaluated with both dose-volume histogram (DVH) and the matched peripheral dose (MPD). Table 2 shows the characteristics of^ 125^I brachytherapy. Patients were placed in a supine position with both arms raised above the head and with the head in a natural position. In order to suppress the movement of respiration, patients were immobilized using a low-density body cradle and the breathing of the patient was repressed by applying thermoplastic material on the abdomen. After target-volume determination, interstitial needles (18-gauge, stainless steel, hollow needles, 15 cm long) were inserted into the tumor, approximately 1 cm apart. CT was used to guide the placement of the needles. Precautions were taken to avoid puncture of large blood vessels (central vein and inferior vena cava). Any bleeding was stopped by application of pressure. A Mick applicator (Radioactive seeds Implantation Instruments, HTA CO., LTD., China) was then sequentially attached to the distal end of each needle to place the ^125^I seeds (Model-6711, HTA CO., LTD., China) into the tumor, spaced approximately 1 cm apart along the needle track. A median of 60 seeds (range, 30-108) was implanted per patient, with a median activity per seed of 0.7 mCi and a median total implanted activity of 22.6 mCi (range, 10-70 mCi). The 83.3% percent of the patients had only 1 site implanted, whereas 16.7% had 2 or 3 sites implanted. The median MPD was 114Gy (range, 60-160Gy). For most implants, more than 50% of the target volume received between 90 and 120 Gy.

**Table 2:** characteristics of ^125^I brachytherapy

Characteristic	Number of patients (%)
Activity per seed	
0.4 mCi	10 (20.8%)
0.7 mCi	23 (47.9%)
1.0 mCi	15 (31.3%)
Number of seeds	
30-40	8 (16.7%)
41-60	21 (43.8%)
≥61	18 (37.5%)
Total activity	
10-20 mCi	10 (20.8%)
21-30 mCi	30 (62.5%)
31-50 mCi	8 (16.7%)
MPD	
60-80 Gy	12 (25%)
90-120 Gy	29 (60.4%)
130-160 Gy	7 (14.6%)
Number of implant sites	
1	40(83.3%)
2	5(10.4%)
3	3(6.25%)

mCi: millicuries; MPD: matched peripheral dose; Gy: gray.

During the treatment, the patients were monitored weekly with physical examination and blood chemistry evaluation. Evaluation of tumor response was based on serial CT scans. All patients had CT scans before initiation of brachytherapy and 4 weeks after completion of radiation therapy and then at 1-3 months intervals. Complete disappearance of hepatic tumor was considered as complete response (CR); decrease of less than 50% of the tumor size as partial response (PR); decrease of more than 50% of the tumor size or no change as stable of disease (SD); and progression as progressive disease (PD). Acute toxicity was evaluated weekly during the treatment and 1 month following the treatment using the Radiation Therapy Oncology Group/European Organization for Research and Treatment of Cancer (RTOG-EORTC) scale. Sub-acute or chronic toxicity was defined as occurring after 1 month. Survival was estimated from the date of diagnosis according to the Kaplan-Meier method. Log rank test was used in the analysis of prognostic factors.

## Results

**Figure 1:**
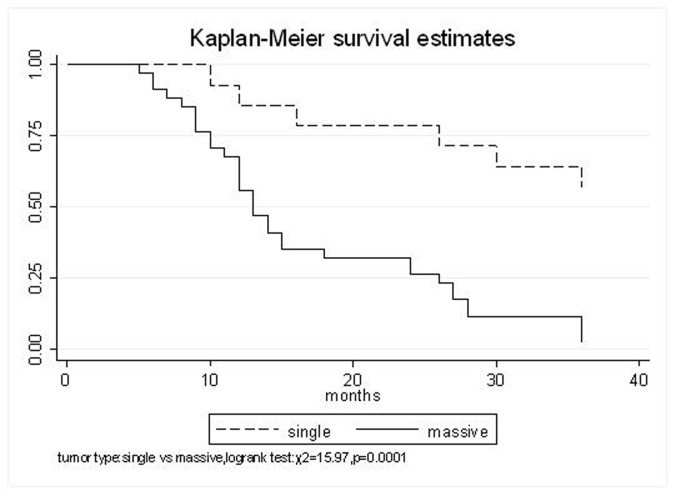
Tumor type, Single VS. Massive

**Figure 2:**
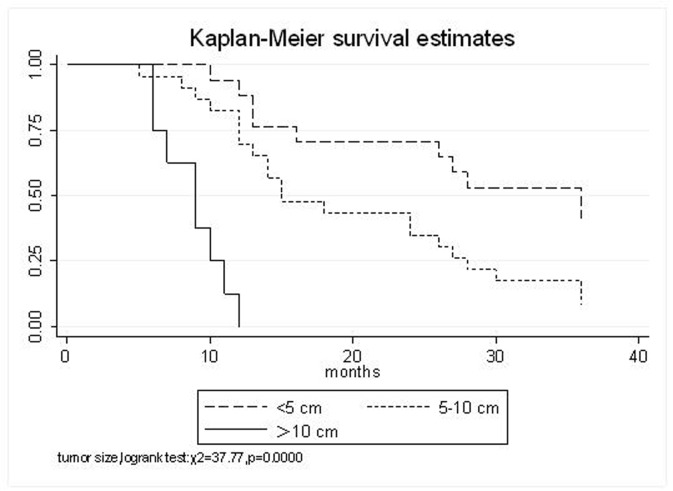
Tumor size, < 5 cm VS. 5-10 cm VS. >10 cm

**Figure 3:**
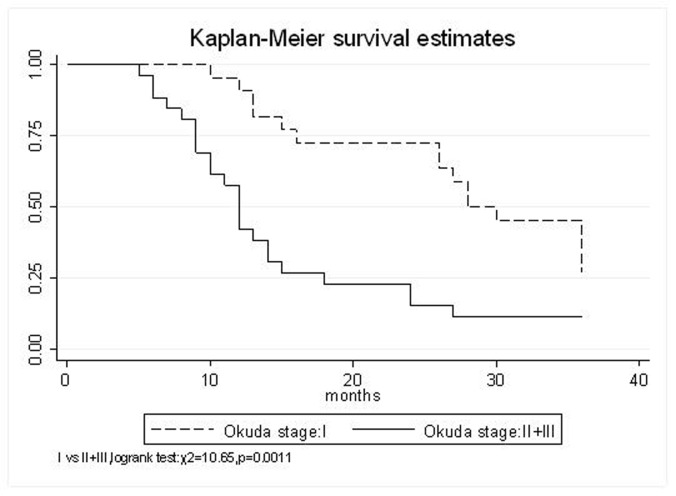
Okuda stage, I VS. II+III

**Figure 4:**
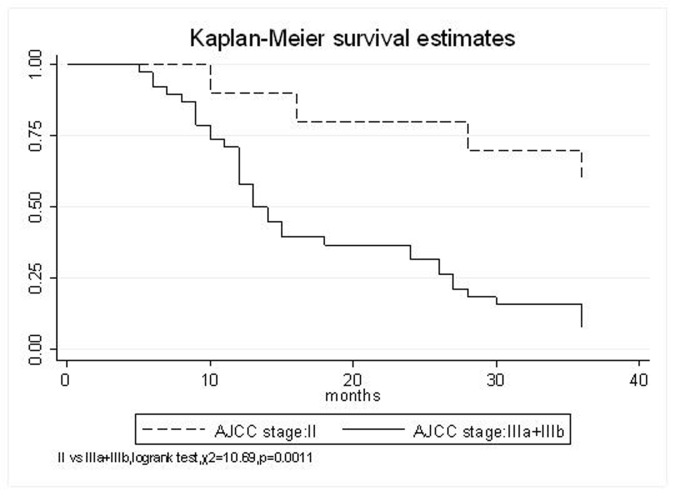
AJCC stage, II VS. IIIa+IIIb

**Figure 5:**
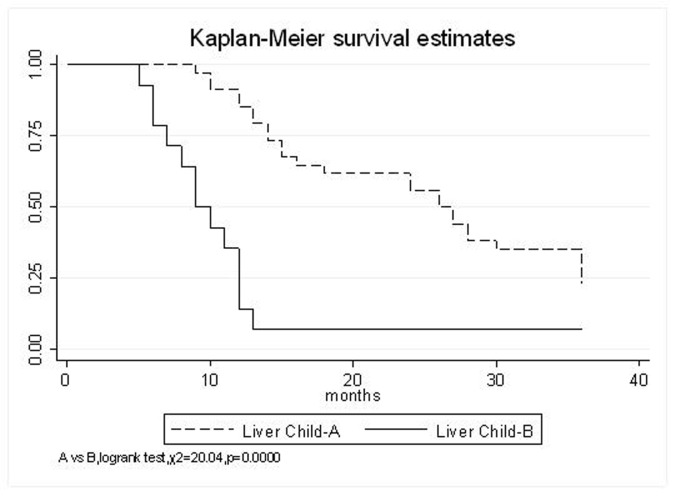
Liver Child-Pugh, A VS. B

**Figure 6:**
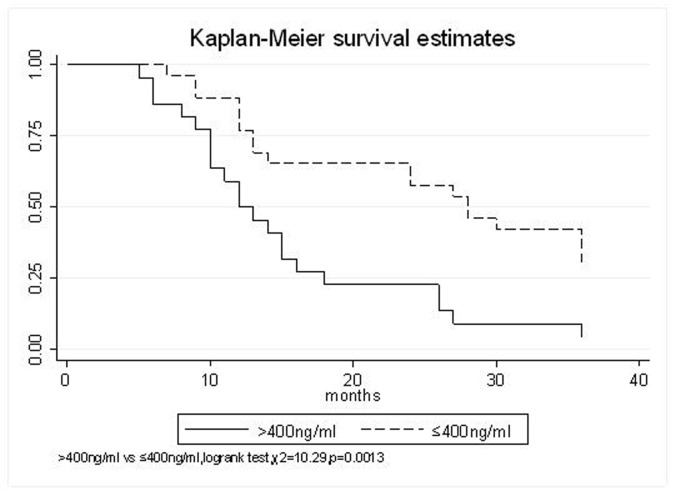
AFP(ng/ml), > 400 VS. ≤ 400

**Figure 7:**
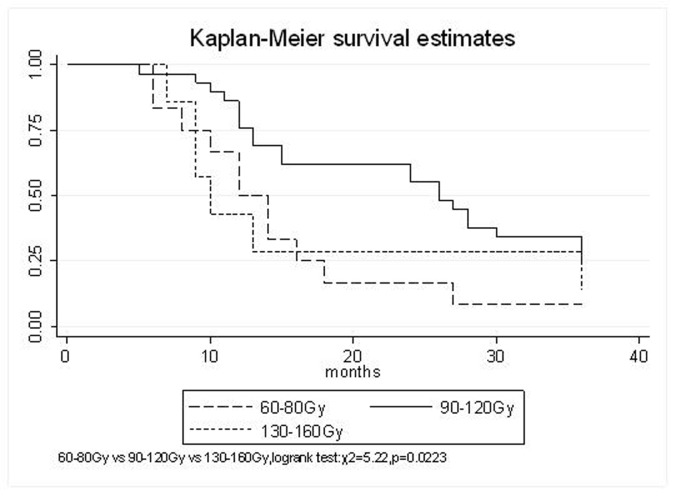
MPD, 60-80 Gy VS. 130-160 Gy VS.90-120 Gy

All patients underwent evaluation of tumor response based on CT scan. Among 48 patients with unresectable hepatic tumors, 8 patients achieved CR and 26 patients achieved PR (Table 3). In total, an objective response was observed in 34 of 48 patients, giving a response rate of 70.8%. At the time of last follow-up in March 2006, 9 patients remained alive and 39 were dead. 16 patients (33.3%) developed intrahepatic metastasis outside the radiation field. Extrahepatic metastasis developed in 7 patients (14.6%), including 3 in lung and 4 in bone. There were no treatment-related deaths. Survival rates were evaluated in all patients from the time of diagnosis. Acute toxicity of ^125^I brachytherapy is summarized in Table 4. The survival rates at 1, 2 and 3 years were 75%, 45.8% and 27.1%, respectively, with a median survival time of 15.5 months (Table 5). Elevation of transaminase was seen in 5 patients (2 patients of grade 1 and 3 of grade 2), bilirubin in 3 patients (1 of grade 1 and 2 of grade 2), albumin in 5 patients (2 of grade 1 and 3 of grade 2) and alkaline phosphatase in 4 patients (2 of grade 1 and 2 of grade 2). Hematologic toxicity included thrombocytopenia in 4 patients (1 of grade 1 and 3 of grade 2), anemia in 7 patients (5 of grade 1 and 2 of grade 2) and leucocytopenia in 6 patients (3 of grade 1 and 3 of grade 2). No patient had radiation-related gastrointestinal bleeding. In the analysis of prognostic factors (Table 6), age, gender, performance status (KPS) did not influence survival significantly (P > 0.05). Tumor type had significant impact on survival rates for single and massive (logrank test, P = 0.0001, Fig. 1). Tumor size had significant impact on survival rates for < 5 cm versus 5-10 cm and versus > 10 cm (logrank test, P = 0.0000, Fig. 2). Okuda stage had significant impact on survival rates for Stage I and Stage II plus III patients (logrank test, P = 0.0011, Fig.3). AJCC stage had significant impact on survival rates for stage II and IIIa+IIIb (logrank test, P=0.0011, Fig.4). Liver Child-Pugh had significant impact on survival rates for Child-A and Child-B (logrank test, P = 0.0000, Fig. 5). Pretreatment AFP levels had a similar impact on survival (logrank test, P = 0.0013, Fig. 6). MPD also had a significant impact on survival (logrank test, P = 0.0223, Fig. 7), indicating MPD 90-120Gy had higher survival rates than which of MPD 60-80Gy and MPD 130-160Gy.

**Table 3:** Response of unresectable hepatic tumor to ^125^I brachytherapy

Response	Number of patients(%)
CR	8 (16.7%)
PR	26 (54.2%)
SD	10 (20.8%)
PD	4 (8.3%)

CR: complete response; PR: partial response; SD: stable disease; PD: progressive disease

**Table 4:** Acute toxicity of ^125^I brachytherapy (n = 48)

	Toxicity grade			
	1	2	3	4
Transaminase	2	3	-	-
Bilirubin	1	2	-	-
Albumin	2	3	-	-
Alkaline phosphatase	2	2	-	-
Leucopenia	3	3	-	-
Anemia	5	2	-	-
Thrombocytopenia	1	3	-	-

**Table 5:** Actuarial overall survival (n = 48)

Category	Number of Patients (%)
Number remaining alive	9 (18.8%)
Death of intrahepatic disease	16 (33.3%)
Death of extrahepatic disease	7 (14.6%)
Death of complications of further Treatment	10 (20.8%)
Death of unrelated cause	6 (12.5%)
Actuarial survival	
1 year	75%
2 year	45.8%
3 year	27.1%
Median survival time	15.5 months (95%CI,12.7-27)

**Table 6:** Actuarial overall survival (n = 48)

Patient group	Survival rate(%)			Mean survival (months)	P
	1-Y	2-Y	3-Y		
All patients	75%	45.8%	27.1%	24.2 (95% CI, 19.2-29.2)	-
Age(years)					
<50	80%	60%	40%	28.9 (95% CI, 14.7-43.1)	0.4683
≥50	73.7%	42.1%	23.7%	22.9 (95% CI, 17.5-28.4)	
Gender					
Male	76.3%	44.7%	26.3%	22.9 (95% CI, 17.7-28.1)	0.5499
Female	70%	50%	30%	29.1 (95% CI, 13.1-45.1)	
KPS					0.3461
100	80%	60%	40%	28.9 (95% CI, 14.7-43.1)	-
80	81%	57.1%	28.6%	26.5 (95% CI, 18.4-34.6)	-
60	64.7%	23.5%	17.6%	18.5 (95% CI, 11.2-25.8)	-
Tumor type					
Single	92.9%	71.4%	64.3%	41.2 (95% CI, 29.8-52.6)	0.0001
Massive	67.6%	32.4%	11.8%	17.1 (95% CI, 13.7-20.6)	
Tumor Size					0.0000
<5 cm	94.1%	70.6%	52.9%	35.5 (95% CI, 25.6-45.5)	
5-10 cm	82.6%	43.5%	17.4%	21.1 (95% CI, 15.6-26.7)	
>10 cm	12.5%	0%	0%	8.75 (95% CI, 6.9-10.6)	
Okuda stage					
I	95.4%	72.7%	45.5%	33 (95% CI, 25.3-40.7)	0.0011
II+III	57.7%	23.1%	11.5%	16.7 (95% CI, 11.3-22.1)	
AJCC stage					
II	90%	80%	70%	41.7 (95% CI, 28-55.4)	0.0011
IIIa+IIIb	71.1%	36.8%	15.8%	19.6 (95% CI, 15.1-24)	
Liver Child–Pugh					
A	85.3%	61.8%	35.3%	9.5 (95% CI, 23.4-35.5)	0.0000
B	35.7%	7.1%	7.1%	11.3 (95% CI, 6.6-16)	
AFP(ng/ml)					
> 400	59.1%	22.7%	9.1%	16.6 (95% CI, 11.1-22.1)	0.0013
≤ 400	88.5%	65.4%	42.3%	30.6 (95% CI, 23.2-38)	
MPD					0.0223
60-80 Gy	66.7%	16.7%	8.3%	15.1 (95% CI, 9.2-21)	
90-120 Gy	86.2%	58.6%	34.5%	29.1(95% CI, 22.3-36)	
130-160 Gy	42.9%	28.6%	28.6%	20.6 (95% CI, 2-39.1)	

## Discussion

^125^I brachytherapy for the treatment of prostate carcinoma has been confessed successful [4-7], however, the sample therapy for HCC is still in research stage. Because of the sensitivity of the normal liver limits the dose that can be delivered, radiotherapy has not been considered for the treatment of HCC for some time. However,^ 125^I seeds brachytherapy is an ideal technique that combines the ability to target tumor cells under direct visualization and spare uninvolved liver parenchyma secondary to the sharp dose fall off outside of the implanted volume. Moreover, because of the liver’s natural regenerative capabilities and its ability to tolerate loss of over 75% of its tissue without ensuing failure, high dose of radiation can be delivered to restricted volumes by brachytherapy. With the property of local “conformal radiotherapy”, normal liver is spared, a potentially tumoricidal dose of radiation (much higher than whole-liver tolerance) can be administered with acceptable complications. TACE has been an effective therapeutic options for treatment of unresectable HCC [8], however, its benefit is limited. So, combination with other adjuvant treatment should be necessary. Our study of 48 patients is unique in that it is the largest series reported to date of ^125^I brachytherapy in the successful treatment of advanced, unresectable HCC, who had failed with TACE, and has the longer follow-up.

The prognostic factors of HCC reported in the literature include tumor size, tumor type, tumor stage, serum AFP status, etc. In our study, tumor type, tumor size, Okuda stage, AJCC stage, liver Child–Pugh, AFP level, and MPD had significant impact on survival. Other known factors were not significant. The significance of the radiation dose has been suggested in terms of induction of tumor regression as well as in overall survival. The best prescription dosage of radioactive ^125^I seed interstitial implantation for HCC and the best radioactivity of seed are still controversial. Ricke J, et al [9-11] thought that the mean minimal dose inside the liver tumor margin amounted to 17-18Gy (range, 10-25Gy); Zhang FJ, et al [12] described that ^125^I seeds of the radioactivity of 30 MBq, MPD was 100 approximately 150 Gy. These reports strongly support the importance of reasonable MPD in inducing tumor regression. In our study, the MPD had significant impact on survival. The survival rates of patients were higher, with MPD 90-120Gy, than which of less than 80Gy or more than 130Gy (χ^2^ = 5.22, P = 0.0223). However, it should be mentioned that the function of the non-tumorous part of the liver might be compromised owing to preexisting parenchymal disease, especially cirrhosis of the liver. Most HCC patients referred for radiotherapy present with advanced unresectable disease, usually associated with cirrhosis of the liver. In the report of the University of Michigan group, 128 patients were treated with conformal hyperfractionated RT delivered with concurrent continuous infusion hepatic arterial FUdR. Thirty-eight patients (30%) developed grade 3-4 toxicity, and 5 cases (4%) of radiation-induced liver disease (RILD) were observed. The median survival of 35 HCC patients was 15.2 months [13, 14]. In our study, no sub-acute or chronic toxicity of grade 3-4 were observed and the median survival was 15.5 months. Nevertheless, the encouraging results did not mean that the occurrence of RILD could be ignored. In our group, the patients with Child-Pugh class C or KPS < 50 were excluded. During the observation, there was neither treatment-related fatal hepatic toxicity nor radiation-related gastrointestinal complication, including gastroduodenal ulcer and bleeding, 34 patients obtained objective response with a response rate of 70.8%. In reports of Zhang FJ et al [12], 37 of the 45 lesions obtained CR or PR, the response rate was 82.2%, no other severe complications, such as massive hemorrhage, bile fistulae, and pancreatic fistula were seen. Using ^125^I brachytherapy in our study, treatment plans according to TPS were designed for each patient, in which the high-dose region encompassed the planning target volume and spared normal tissues. Our study had no treatment-related deaths.

In conclusion, permanent ^125^I brachytherapy induced a substantial tumor response rate of 70.8% with survival rates at 1, 2 and 3 years of 75%, 45.8% and 27.1%, respectively, and a median survival time of 15.5 months in patients with unresectable HCC who had failed TACE. Patients with massive tumor, tumor size ≥ 5cm, Okuda stage II/III, AJCC stage III, Liver Child–Pugh B, pretreatment AFP level of >400 ng/ml, and MPD ≤ 80Gy or ≥ 130Gy had significantly shorter survival. With the property of local “conformal radiotherapy” and the advantages of minimal invasion, convenience, high performance, slight adverse effect, permanent ^125^I seeds implantation is a safe and effective adjuvant treatment for unresectable HCC. The complications are acceptable and can be managed with conservative treatment. Although we do not know whether there is a long-term survival benefit through the use of this treatment, permanent ^125^I brachytherapy seems to be an important alternative to other locally ablative techniques for this subset of patients. Further study is warranted to evaluate the survival of such patients with controlled trials.
